# MicroRNA profiling of a CD133^+^ spheroid-forming subpopulation of the OVCAR3 human ovarian cancer cell line

**DOI:** 10.1186/1755-8794-5-18

**Published:** 2012-05-29

**Authors:** Eun Ji Nam, Maria Lee, Ga Won Yim, Jae Hoon Kim, Sunghoon Kim, Sang Wun Kim, Young Tae Kim

**Affiliations:** 1 Institute of Women’s Life Medical Science, Women’s Cancer Clinic, Department of Obstetrics and Gynecology, Yonsei University College of Medicine, Seoul, South Korea; 2 Department of Obstetrics and Gynecology, Yonsei University College of Medicine, Seongsan-no 250, Seodaemun-gu, C.P.O. Box 8044, Seoul, South Korea, 120-752

**Keywords:** MicroRNA, Cancer stem cell, Ovarian cancer, CD133, OVCAR3, Chemoresistance

## Abstract

**Background:**

Cancer stem cells (CSCs) are thought to be a source of tumor recurrence due to their stem cell-like properties. MicroRNAs (miRNAs) regulate both normal stem cells and CSCs, and dysregulation of miRNAs has an important role in tumorigenesis. Cluster of differentiation (CD) 133^+^ and spheroid formation have been reported to be one of the main features of ovarian CSCs. Therefore, we determined the miRNA expression profile of a CD133^+^ spheroid-forming subpopulation of the OVCAR3 human ovarian cancer cell line.

**Methods:**

Initially, we confirmed the enrichment of the OVCAR3 CD133 subpopulation by evaluating *in vitro* anchorage-independent growth. After obtaining a subpopulation of CD133^+^ OVCAR3 cells with > 98% purity via cell sorting, miRNA microarray and real-time reverse transcription-polymerase chain reaction (RT-PCR) were performed to evaluate its miRNA profile.

**Results:**

We found 37 differentially expressed miRNAs in the CD133^+^ spheroid-forming subpopulation of OVCAR3 cells, 34 of which were significantly up-regulated, including *miR-205, miR-146a, miR-200a, miR-200b*, and *miR-3,* and 3 of which were significantly down-regulated, including *miR-1202* and *miR-1181*.

**Conclusions:**

Our results indicate that dysregulation of miRNA may play a role in the stem cell-like properties of ovarian CSCs.

## Background

Epithelial ovarian cancer (EOC) is the most lethal of all gynecologic malignancies. Although an initial treatment method offers a response rate of 70%, most patients eventually relapse with chemo-resistant disease [1]. Thus, identification of new molecular markers that target chemo-resistant disease is needed for therapeutic approaches to ovarian cancer.

Cancer stem cells (CSCs) have recently been determined to comprise a small proportion of highly malignant cancer cells possessing stem cell properties [2]. It is generally accepted that stem cells are more resistant to apoptosis and DNA damage and are therefore more likely to be resistant to chemotherapy [3]. As such, if CSCs are refractory to therapy, they are unlikely to be curative and relapse would be expected. Previous studies of acute myelogenous leukemia (AML) provided compelling evidence of the existence of CSCs [4]. Thereafter, several studies characterized CSCs in other types of tumors including those of the brain, breast, colon, pancreas, prostate, and ovaries.

Self-renewal and lineage capacity are the hallmarks of any stem cell [2]. Therefore, a few methods have been developed to use these characteristics in order to assay cancer stem cells. The ability of cells to form tumor spheres is one such method, evaluating the capacity of cancer cells to grow as multi-cellular spheroids under non-differentiating and non-adherent conditions [5]. Using this method, ovarian CSCs from patients with ascites are first isolated and their ability to exhibit stem cell-like properties is examined [6]. In addition, *in vivo* serial transplantation assays, dye exclusion assays, and isolation via cell surface specific antigen profile methods are now used to identify CSCs. In ovarian cancer, the most commonly used cell surface marker to identify ovarian CSCs involves the use of cluster of differentiation (CD) 133^+^ cell populations [7].

MicroRNAs (miRNAs) are 21–23 nucleotides long and act as regulatory molecules by either inhibiting translation or promoting degradation of target mRNA transcripts [8]. MiRNA-driven pathways are fundamental for the maintenance and proper function of cell stemness in embryonic stem cells. Biologically significant miRNA-driven pathways in embryonic stem cells have also been identified in CSCs and are speculated to be involved in oncogenesis [9]. Recently, *miR-200a* was reported to be down-regulated in CD133/1+ ovarian cancer stem cells [10]. Likewise, CD44^+^ epithelial ovarian CSCs were reported to have low levels of *miR-199a* and *miR-214*[11]. Guo and colleagues reported that the expression levels of *miR-204, miR-206, miR-223, miR-9, miR-100,* and *miR-200c* were dysregulated in CD133^+^ OVCAR3 human ovarian cancer cells [12]. However, only limited data are available regarding miRNA expression profiles of ovarian CSCs.

In this study, miRNA expression profiles of a CD133^+^ spheroid-forming subpopulation of OVCAR3 ovarian cancer cells were investigated to identify miRNA expression profiles that contribute toward the characteristics of CSCs in ovarian cancer.

## Results

### Determination of the capacity of ovarian cancer cell lines for *in vitro* anchorage-independent growth

**Figure 1:**
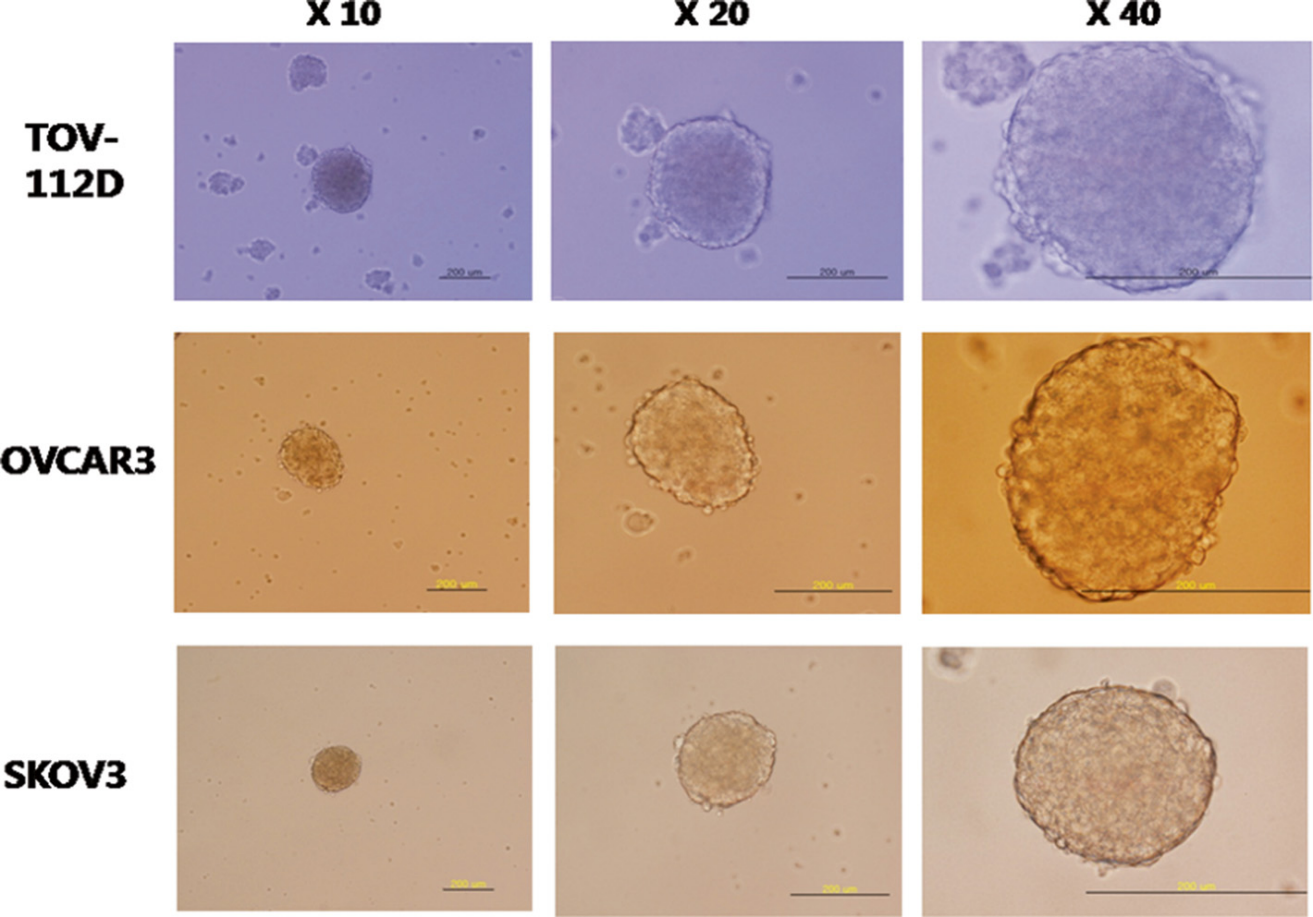
Representative images of tumor spheres from OVCAR3, TOV112D, and SKOV3 cells in a sphere culture system with serum-free DMEM-F12 (Invitrogen, Carlsbad, Calif., USA) supplemented with 10 ng/mL basic fibroblast growth factor (bFGF) and 20 ng/mL epidermal growth factor (EGF) and plated in an ultra-low attachment plate.

We first tested the capacity for anchorage-independent growth, and tumor spheroid formation was noted in OVCAR3, TOV112D, and SKOV3 (Figure 1). OVCAR433B and OVCAR429 did not make tumor spheroids in the non-adherent culture system. Stringent, low-density culture systems gave rise to tumor sphere formation. Tumor spheres tend to grow within a week as multi-cellular spheroids under non-differentiating and non-adherent conditions, with the numbers of tumor spheres reaching a maximum at two weeks. Tumor spheres are small, non-adherent, compact, and non-symmetric, and primary spheres can be enzymatically dissociated to single cells, which in turn give rise to secondary spheres.

### Increased paclitaxel resistance of ovarian cancer cells in tumor spheroids

**Figure 2:**
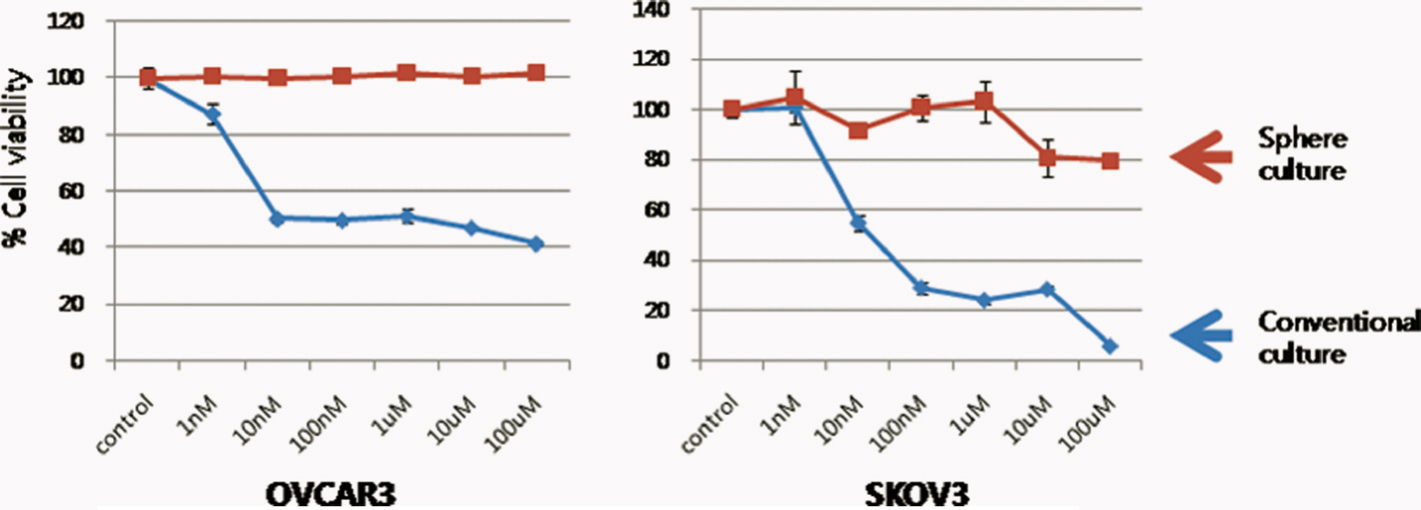
Increased paclitaxel resistance of OVCAR3 and SKOV3 cells in an anchorage-independent culture system compared with those in a conventional adherent culture system.

To evaluate whether tumor spheres demonstrate increased resistance to chemotherapy, we compared cell viabilities between OVCAR3 and SKOV3 cells in a conventional adherent culture system and tumor spheroids using MTT assay. Spheroids are known to contain a greater number of CSCs [6], and in accordance with previous observations, increased paclitaxel resistance was noted in spherical ovarian cancer cells (Figure 2). 

### OVCAR3 cells in anchorage-independent culture are enriched with CD133^+^

**Figure 3:**
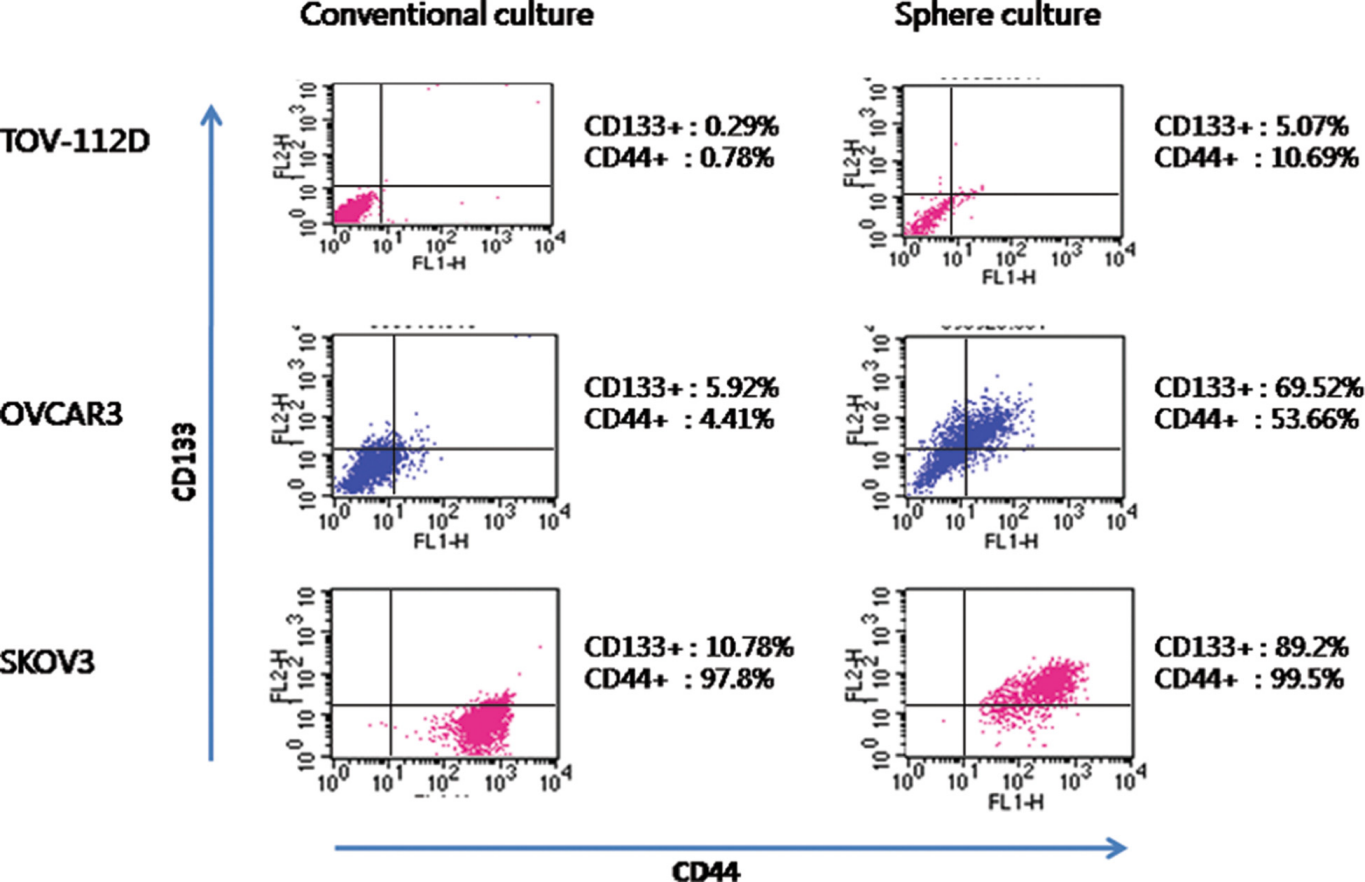
**Flow cytometry analysis with OVCAR3, SKOV3, and TOV-112D were performed before and after anchorage-independent culture at least three times. CD133**^**+**^**and CD44**^**+**^**populations were enriched in a sphere culture system in TOV112D and OVCAR3 cells; however, CD44**^**+**^**populations were initially high in the SKOV3 cell line.**

We determined the positivity of two CSC markers - CD133 and CD44, which are known as ovarian CSC markers (Figure 3). We found that CD133^+^ and CD44^+^ populations were enriched in tumor spheroids from OVCAR3 and TOV112D cells. However, the CD44^+^ subpopulation presented major proportion in SKOV3 cells under both conventional culture conditions and tumor spheroids. Because the CD133^+^ subpopulation was the most efficiently enriched in OVCAR3 tumor spheroids, we then attempted to document the differential expression of miRNAs in CD133^+^ sphere forming subpopulations in OVCAR3 cells. The average CD133 positivity of OVCAR3 cells in a conventional adherent culture system was 6.07 ± 2.31% while that of OVCAR3 cells in tumor spheres was 74.02 ± 5.50% (*P* = 0.047).

### The CD133^+^ spheroid forming subpopulation of the OVCAR3 human ovarian cancer cell line over-expresses ‘stemness’ genes

Although we found that the CD133^+^ subpopulation was enriched in tumor spheroids, the spheroids themselves were comprised of various kinds of cells. Indeed, the average purity of CD133^+^ cells in tumor spheroids of OVCAR3 was 74.02 ± 5.50% according to flow cytometric analysis. Therefore, we performed cell sorting with a CD133 antibody in order to obtain a more purified subpopulation of CD133^+^ cells from tumor spheres of OVCAR3 cells. We then tested the expression levels of genes that correlate with “stemness,” namely Oct-4, Sox-2, and Nanog, in cancer cells from adherent culture systems, as well as CD133^+^ and CD133^-^ cells from tumor spheres.

**Figure 4:**
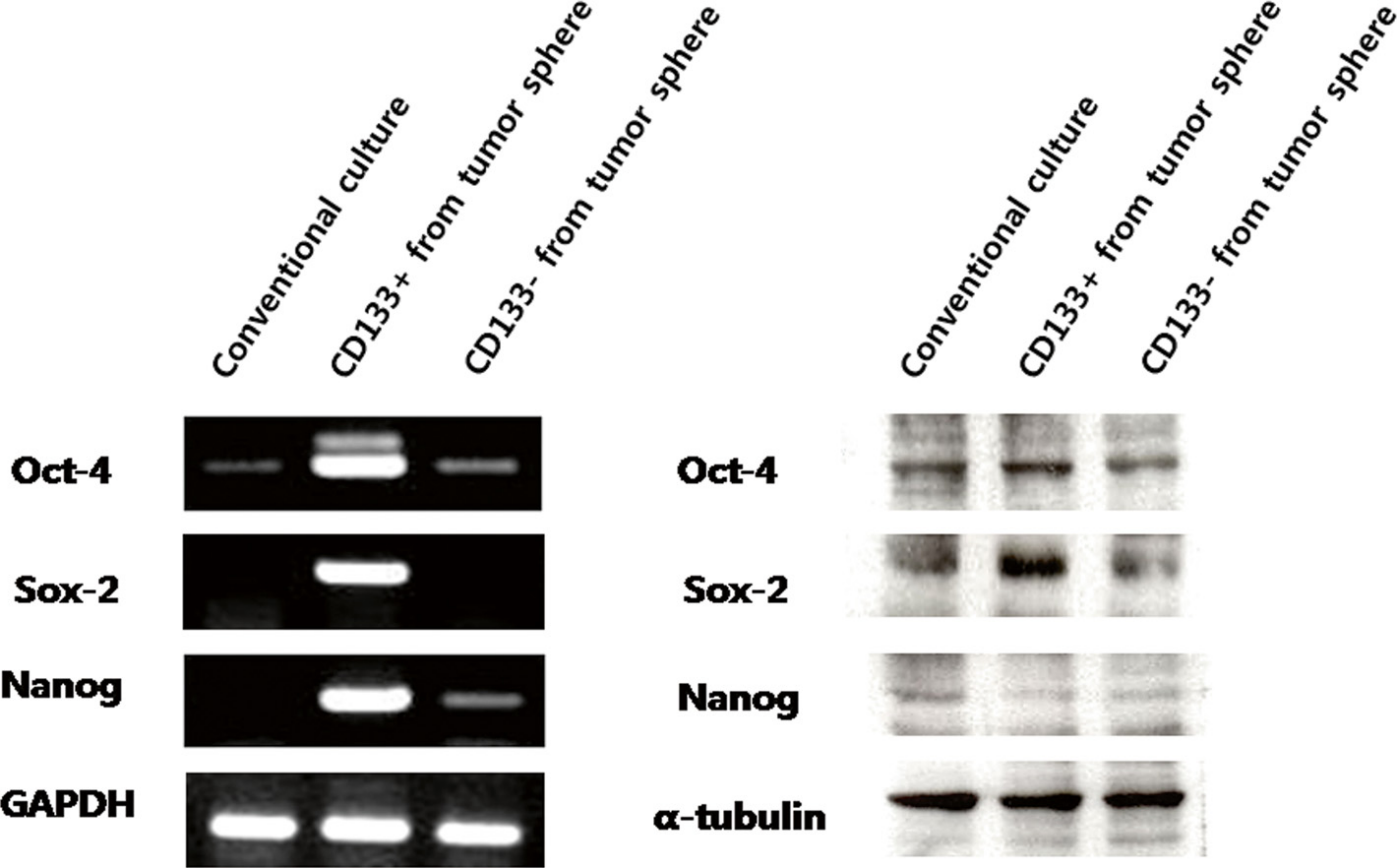
**RT-PCR and Western blot analysis showed that a CD133**^**+**^**spheroid-forming subpopulation of OVCAR3 over-expresses “stemness” genes, compared to the levels of cancer cells from an adherent culture system as well as those from a CD133**^**-**^**spheroid-forming subpopulation of OVCAR3 cells.**

RT-PCR analysis revealed that the mRNA expressions of Oct-4, Sox-2, and Nanog were significantly increased in the CD133^+^ tumor spheroid-forming subpopulation of OVCAR3 cells, compared with those in cancer cells from CD133^-^ adherent cultured cells as well as those in the CD133^-^ tumor spheroid-forming subpopulation of OVCAR3 cells (Figure 4). In accordance with RT-PCR results, Western blot analysis showed that the protein expressions of Oct-4 and Sox-2 were up-regulated in the CD133^+^ tumor spheroid-forming subpopulation, while the protein level of Nanog was not increased. Based on these results, we determined that the CD133^+^ spheroid-forming population of OVCAR3 cells over-expresses “stemness” genes that may be associated with stem cell-like characteristics.

RT-PCR analysis revealed that the mRNA expressions of Oct-4, Sox-2, and Nanog were significantly increased in the CD133^+^ tumor spheroid-forming subpopulation of OVCAR3 cells, compared with those in cancer cells from CD133^-^ adherent cultured cells as well as those in the CD133^-^ tumor spheroid-forming subpopulation of OVCAR3 cells (Figure 4). In accordance with RT-PCR results, Western blot analysis showed that the protein expressions of Oct-4 and Sox-2 were up-regulated in the CD133^+^ tumor spheroid-forming subpopulation, while the protein level of Nanog was not increased. Based on these results, we determined that the CD133^+^ spheroid-forming population of OVCAR3 cells over-expresses “stemness” genes that may be associated with stem cell-like characteristics.

### MicroRNA microarray analysis

Based on the RT-PCR and Western blot analysis results, we compared the miRNA expression profiles between the OVCAR3 CD133^+^ spheroid-forming subpopulation and OVCAR3 cells from an adherent culture system. After cell sorting using FACSaria, the purity of this sorted population as assessed using post-sort flow cytometry was > 98% for the CD133^+^ fraction. Then, we extracted miRNAs and performed miRNA microarray analysis.

**Figure 5:**
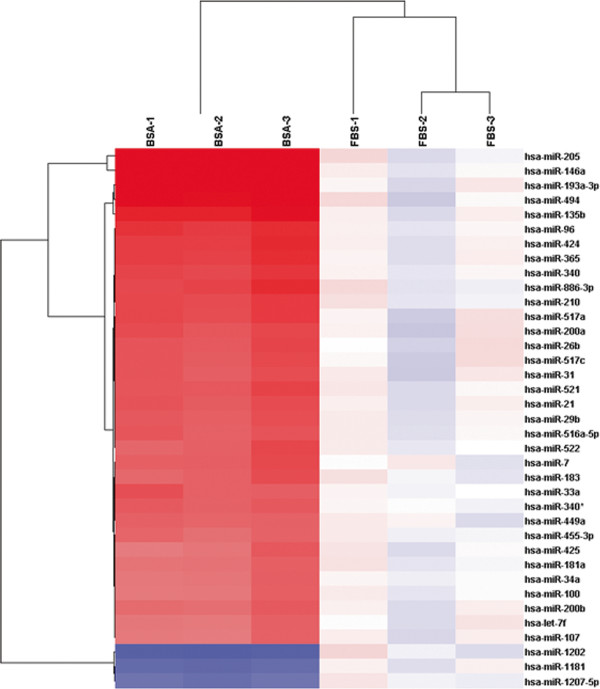
Unsupervised hierarchical clustering analysis of miRNAs that exhibited a > two-fold increase or decrease in the CD133+ spheroid-forming subpopulation of the OVCAR3 human ovarian cancer cell line.

We identified a total of 37 miRNAs that were differentially expressed between the CD133^+^ spheroid-forming subpopulation of OVCAR3 cells and those grown in adherent cell culture. Thirty-four miRNAs including *miR-205, miR-146a, miR-200a, miR-200b,* and *miR-31* were significantly up-regulated, while 3 microRNAs including *miR-1202* and *miR-1181* were significantly down-regulated (Figure 5, Table 1).

**Table 1:** Differentially expressed miRNAs with > 2-fold change in a CD133^+^ spheroid forming subpopulation versus cancer cells with conventional adherent culture conditions

**miRNA**	**Welch*t*-test** ***P*-value**	**Fold change**
hsa-miR-205	0.00127	11.7804
hsa-miR-146a	0.000693	7.33415
hsa-miR-193a-3p	0.00384	4.31891
hsa-miR-494	0.00879	3.94522
hsa-miR-135b	0.00368	3.39828
hsa-miR-96	0.00403	2.89271
hsa-miR-424	0.00456	2.83982
hsa-miR-365	0.00563	2.79782
hsa-miR-886-3p	0.00261	2.72994
hsa-miR-340	0.0029	2.66247
hsa-miR-210	0.00385	2.61775
hsa-miR-517a	0.0196	2.60692
hsa-miR-200a	0.0265	2.45554
hsa-miR-521	0.00856	2.42654
hsa-miR-26b	0.0207	2.41395
hsa-miR-517c	0.0254	2.38547
hsa-miR-21	0.013	2.37583
hsa-miR-31	0.0247	2.34642
hsa-miR-7	0.000204	2.32079
hsa-miR-29b	0.01	2.31852
hsa-miR-522	0.00152	2.29752
hsa-miR-516a-5p	0.0101	2.29596
hsa-miR-33a	0.0255	2.28197
hsa-miR-183	0.000939	2.25637
hsa-miR-340*	2.43E-05	2.2503
hsa-miR-449a	0.00276	2.1729
hsa-miR-200b	0.0143	2.13023
hsa-miR-455-3p	0.00331	2.10618
hsa-miR-425	0.00889	2.05804
hsa-miR-181a	0.00688	2.05351
hsa-miR-34a	0.00155	2.0393
hsa-miR-100	0.00509	2.02263
hsa-let-7f	0.0193	2.01977
hsa-miR-107	0.0182	2.00025
hsa-miR-1202	0.0134	0.41997
hsa-miR-1181	0.023	0.47041
hsa-miR-1207-5p	0.00905	0.49464

### Validation of microRNA results

To validate the microarray results, qRT-PCR was performed with seven miRNAs that exhibited a > two-fold change in expression (Table 2). In agreement with the microarray results, *miR-205, miR-146a, miR-200a, miR-200b,* and *miR-31* were up-regulated, whereas *miR-1202* and *miR-1181* were down-regulated in the CD133^+^ spheroid-forming subpopulation (Table 2). Overall, the microarray data were considered sufficient to warrant further analyses in a clinical setting.

**Table 2:** Validation of miRNA microarray results using quantitative RT-PCR: miRNAs differentially expressed between OVCAR3 cells from the CD133^+^ sphere-forming subpopulation compared with cells cultured in a conventional adherent environment

	**MicroRNA microarray**		**qRT-PCR**
	**Fold change**	***P*value**	**Fold change (mean ± SD)**
has-miR-205	11.78	0.001	11.76 ± 1.32
has-miR-146a	7.33	<0.001	5.19 ± 4.14
has-miR-200a	2.45	0.0265	2.21 ± 2.54
has-miR-200b	2.13	0.014	1.81 ± 0.54
has-miR-31	2.35	0.024	2.45 ± 1.59
has-miR-1202	0.41	0.013	0.58 ± 0.22
has-miR-1181	0.47	0.023	0.67 ± 0.28

## Discussion

This study showed that a CD133^+^ sphere-forming subpopulation of OVCAR3 cells had a distinct microRNA expression profile compared with that of OVCAR3 cells grown in adherent culture conditions. These findings might contribute to understanding miRNA-driven pathways related to chemoresistance in ovarian cancer.

Initially, we tested whether various ovarian cancer cell lines can make tumor sphere. Interestingly, tumor spheres were observed in OVCAR3, SKOV3, and TOV112D cells. Previous studies revealed that a small subpopulation of CSCs exists within tumor spheres [13]. MTT assay showed that tumor spheres from the OVCAR3 and SKOV3 cells were resistant to paclitaxel. Given these results, we may speculate that tumor spheres may contain an increased proportion of CSCs. On the other hand, there is a possibility that these findings could also be partly explained by the phenomenon of a worse drug penetration into the inner layer of the spheroid as compared to monolayer cultures. Therefore, we tested whether spheroid-forming cells express stem cell-associated surface markers. Reproducible isolation using distinct cell surface antigens is now regarded as a required characteristic of CSCs [14]. Importantly, enrichment of the cell surface marker CD133^+^ was observed in tumor spheres, which shows that spheroids contain more CSCs than adherent cultures. However, CD44^+^ cells were initially high in the SKOV3 cell line in the conventional culture system. Curley and colleagues showed a relatively high tumorigenic potential of CD133^+^ cells derived from primary human ovarian tumors in an *in vivo* serial transplantation model [7]. Likewise, Ferrandina and colleagues reported that CD133^+^ ovarian tumor cells exhibited higher clonogenic efficiency compared with that of CD133^-^ cells [15]. Therefore, these results suggest that CD133 may be a good marker for identifying ovarian CSC populations *in vitro*. Then, for miRNA expression analysis, we picked the OVCAR3 cell line because the CD133^+^ subpopulation was the most efficiently enriched in OVCAR3 cells with the sphere culture system.

Then, the expression of genes specific to embryonic stem cells was examined in spheroids. Poorly differentiated tumors exhibit over-expression of genes that are normally enriched in embryonic stem cells [16]. We measured the expressions of the Oct-4, Sox-2, and Nanog using RT-PCR and Western blot analysis. Intriguingly, the over-expressions of stem cell-related genes were noted in CD133^+^ spheroid-forming populations of OVCAR3 cells, but not in a CD133^-^ spheroid-forming subpopulation or in cancer cells grown in adherent culture conditions. These results suggest that only CD133^+^ spheroid-forming cells are associated with the expressions of stem cell-related genes and presumably have stem cell-like properties.

Therefore, we sorted CD133^+^ cells from tumor spheres using a FACSAria flow cytometer for miRNA analysis. In this study, we identified 37 miRNAs that were differentially expressed in CD133^+^ spheroid-forming cells compared with the levels in cancer cells in conventional adherent culture conditions of OVCAR3. These miRNAs may be associated with stem cell-like characteristics of ovarian cancer stem cells. While our manuscript was under review, a study of miRNA expression profiling the CD133^+^ subpopulation of OVCAR3 was published by Guo and colleagues [12]. They found that the expressions of 40 miRNAs were > two-fold higher and those of 112 miRNAs were > two-fold lower in CD133^+^ cells compared to those in CD133^-^ cells. They showed up-regulation of the expressions of *miR-204* and *miR-206* and down-regulation of *miR-9, miR-100, miR-223,* and *miR-200c* in CD133^+^ cells using RT PCR. However, their results were not consistent with our miRNA expression profiling results.

We speculate that this contradiction may be explained by differences in the methods used to expand the CSC population prior to sorting. We enriched CD133^+^ cells using a tumor sphere assay; however, Guo and colleagues sorted CD133 cells using FACS only. Differences in culture conditions may have also affected miRNA microarray results. Ince and colleagues used differential culture methods to isolate two separate cell populations from the same initial source [17]. The two populations had differential tumorigenic potential following injection into immunocompromised mice and generated histologically different tumors with distinct metastatic potential. Taken together, these data suggest that the characteristics of putative CSCs may vary depending on the techniques used to isolate the CSCs. Indeed, it is becoming evident that more stringent and clear methodologies for the isolation of CSCs should be developed.

The limitation of this study is that only one cell line-OVCAR3 was used to analyze miRNA expression patterns for CD133^+^ spheroid forming subpopulations. Therefore, we should note that differential expression patterns of miRNAs in CSCs of OVCAR3 cells in this study are not applicable for ovarian cancer in general. However, it is worth noting the reason why only one cell line was chosen for miRNA analysis. During this work, we found that various cell lines from single organ ovary could have differential stem cell characteristics.

In the present study, *miR-205* was present at a level of approximately 10-fold higher in a CD133^+^ spheroid-forming subpopulation of OVCAR3 cells compared with the levels in cells in the adherent culture condition. Recent studies have demonstrated that *miR-205* has a role in both normal and cancer development, but these results are controversial. Specifically, the expression of *miR-205* is significantly suppressed in ovarian cancer [18], whereas *miR-205* is significantly up-regulated in bladder cancer [19]. Inhibition of the Src family can cause cell cycle arrest and growth suppression of ovarian cancer cells, which may paradoxically result in chemo-resistant characteristics of ovarian CSCs. Additionally, *miR-205* was found to be highly expressed in stem cell-rich populations and thus may have a function in normal mammary stem cell maintenance [20]. However, down-regulation of miR-205 was noted in prostate cancer cell lines resistant to chemotherapy [21].

*MiR-146a* was second most differentially expressed (7.33 fold) in CD133^+^ spheroid-forming subpopulations of OVCAR3 cells. This miRNA is mainly expressed in primitive hematopoietic stem cells and T lymphocytes [22]. Reduced expression of *miR-146a* in pancreatic cancer cells was observed compared with the level in normal human pancreatic duct epithelial cells [23]. On the other hand, higher expression of *miR-146a* in NK/T cell lymphoma demonstrated better prognosis [24]. Taken together, further studies are needed to validate whether these miRNAs are associated with chemo-resistance and regulation of CSC function.

## Conclusions

In conclusion, our results indicate that dysregulation of miRNA may play a role in the stem cell-like properties of ovarian CSCs. We are currently working to determine the possible role of individual miRNAs in chemoresistance, which will provide insight into the relevant molecular targets for therapeutic intervention and the eventual development of more intelligent treatment strategies for chemo-resistant ovarian cancer.

## Methods

The experimental protocol was approved by the 85 Institutional Review Board of the Yonsei University College of Medicine (4-2010-0475).

### Ovarian cancer cell line

The human epithelial ovarian cancer cell lines OVCAR3, SKOV3, TOV112D, OVCAR443B, and OVCAR429 were obtained from the Korean Cell Line Bank. Ovarian cancer cells were maintained in MEM (Life Technologies, Inc., Grand Island, NY, USA) supplemented with 10% fetal bovine serum and 100 μg/ml streptomycin in a humidified 5% CO_2_ incubator.

### Assessment of the capacity for *in vitro* anchorage-independent growth

In order to assess spheroid formation, approximately 5 x 10^3^ cells were suspended in 10 mL of serum-free DMEM-F12 (Invitrogen, Carlsbad, Calif., USA), supplemented with 10 ng/mL basic fibroblast growth factor (bFGF), 20 ng/mL epidermal growth factor (EGF) and plated in an ultra-low attachment plate (Corning Incorporated Life Sciences, MA, USA) to prevent adherence. Spheres were counted at 14 days after plating. All experiments were performed in triplicate.

### MTT assay

The 3-(4,5-dimethylthiazol-2-yl)-2,5-diphenyltetrazolium bromide (MTT) assay was performed as previously described [25,26]. Briefly, cells (2 x 10^3^ cells in 100 uL per well) were plated in 96-well plates (flat-bottomed plate, Nunc, Naperville, IL, USA) and incubated with serially diluted paclitaxel containing media. Cell viabilities were determined after 48, 72, and 96 hours.

### FACS

Cells were separated by treatment with 0.25% trypsin/EDTA (Invitrogen, Carlsbad, Calif., USA) and washed once with PBS. After incubation with anti-CD133-PE (Miltenyi Biotech, Auburn, CA, USA) and anti-CD44-FITC (eBioscience, Inc., a San Diego, USA) for 30 min on ice in the dark, the cells were washed twice with 1 ml of ice-cold FACS buffer and centrifuged (400x *g*) for 5 min at 4°C. Cells were then re-suspended in 5 ml of PBS with 2% fetal bovine and analyzed on a FACSCalibur flow cytometer. Further sorting of the CD133^+^ subpopulation of cells was performed using FACSaria (BD FACSaria II special order system, Biosciences, NJ, USA). All flow cytometry results were obtained from two independent experiments with samples performed in triplicate.

### Reverse transcription-PCR and quantitative real-time RT-PCR

Total RNAs were extracted from cells using an RNA Mini Kit (Qiagen), reverse-transcribed into cDNAs, amplified for 30 cycles in 20 μL reactions with primers (Additional file 1: Table S1), and PCR products were electrophoresed on 2% agarose gels using glyceraldehydes 3-phosphate dehydrogenase (GAPDH) as a loading control.

For quantitative real-time reverse transcription-polymerase chain reaction (qRT-PCR), we isolated total miRNAs from cells according to previously described methods [27] using a mirVANA microRNA Isolation Kit (Ambion, Austin, TX, USA) according to the manufacturer’s protocol. Then, cDNAs were synthesized using TaqMan MicroRNA Assays (Applied Biosystems, Foster City, CA, USA). RT-PCR was performed using an Applied Biosystems Prism 7500 Fast Sequence Detection System (Applied Biosystems, Warrington, UK) according to the manufacture’s protocol. All experiments were repeated three times.

### Protein isolation and western blot analysis

Sorted cells were lysed in a lysis buffer containing 50 mM Tris·HCl (pH 7.4), 150 mM NaCl, and 1% Triton X-100. Proteins were separated using 10% sodium dodecyl sulfate-polyacrylamide gel electrophoresis (SDS-PAGE) under denaturing conditions and were transferred to nitrocellulose membranes. The membranes were incubated with anti-Sox2 (1:250; Abcam, Cambridge, MA, USA), anti-Oct-4 (1:1000: Cell Signaling Technology, Inc, USA), anti-Nanog (1:2000; Cell Signaling Technology, Inc., USA), and anti-tubulin primary antibodies (1:2000 Abcam, Cambridge, MA, USA) followed by either an anti-rabbit or anti-mouse secondary antibody conjugated with horseradish peroxidase (1:5,000; Abcam, Cambridge, MA, USA). Antigen-antibody complexes were detected with an ECL chemiluminescence detection system (Amersham, Arlington Heights, IL, USA).

### MicroRNA microarray analysis

A total RNA sample (100 ng) containing miRNA was labeled with Cyanine 3-pGp (Cy3) using an Agilent miRNA Complete Labeling and Hyb Kit (Agilent Technologies, CA, USA). The sample was placed on an Agilent Human miRNA v14 chip (AMDID 029297) and covered with a Gasket slide (Agilent Technologies, CA, USA). Slides were hybridized for 16 hours at 42°C in an Agilent hybridization system. MicroRNA arrays were analyzed using GeneSpring GX v11.5 (Agilent Technologies, CA, USA) with the standard normalization method for one-channel microarrays with the percentile median normalization method. Fold-change values were calculated for unpaired comparisons between normal and test samples and then averaged to generate a mean fold-change value. Welch's *t*-test was used to determine significant changes. Target predictions of significantly changed miRNAs were analyzed using TargetScan 5.1 and miRBase v16 databases.

### Statistical analysis

All experiments were carried out in triplicate. Data are expressed as mean ± standard deviation of at least three independent experiments. The significance of differences was determined using Student’s *t-*test (Version 18.0, SPSS, Inc., USA).

## Competing interests

The authors have no conflicts of interest to declare.

## Authors' contributions

EJ Nam and YT Kim conceived of the study, and participated in its design and coordination and helped to draft the manuscript.

GW Yim, JH Kim, S Kim, and SW Kim carried out molecular genetic studies and participated in the design of the study. All authors read and approved the final manuscript

## Pre-publication history

The pre-publication history for this paper can be accessed here:

http://www.biomedcentral.com/1755-8794/5/18/prepub

## Supplementary Material

Additional file 1 Table S1. PCR Primers. Click here for file
