# Supplementary material for: MicroRNA profiling of a CD133+ spheroid-forming subpopulation of the OVCAR3 human ovarian cancer cell line
Source: BMC Med Genomics. 2012 May 29;5:18. doi: 10.1186/1755-8794-5-18 (PMC3480901; doi:10.1186/1755-8794-5-18)
Supplement: Additional file 1 Table S1. — PCR Primers. [file 1755-8794-5-18-S1.doc]

**Supplementary Table 1.** PCR Primers

| Gene | Forward Primer Reverse Primer | |
| --- | --- | --- |
| GAPDH | GAAGGTGAAGGTCGGAGTC | GAAGATGGTGATGGGATTTC |
| Oct4 | AGTGAGAGGCAACCTGGAGA | CAAAAACCCTGGCACAAACT |
| Nanog | AAGACAAGGTCCCGGTCAAG | CCTAGTGGTCTGCTGTATTAC |
| SOX2 | AGAACCCCAAGATGCACAAC | ATGTAGGTCTGCGAGCTGGT |
